# Long Non-coding RNA H19 Induces Cerebral Ischemia Reperfusion Injury via Activation of Autophagy

**DOI:** 10.14336/AD.2016.0530

**Published:** 2017-02-01

**Authors:** Jue Wang, Bin Cao, Dong Han, Miao Sun, Juan Feng

**Affiliations:** Department of Neurology, Shengjing Hospital, Affiliated Hospital of China Medical University, Shen Yang, 110004, China; Department of Neurology, Shengjing Hospital, Affiliated Hospital of China Medical University, Shen Yang, 110004, China; Department of Neurology, Shengjing Hospital, Affiliated Hospital of China Medical University, Shen Yang, 110004, China; Department of Neurology, Shengjing Hospital, Affiliated Hospital of China Medical University, Shen Yang, 110004, China; Department of Neurology, Shengjing Hospital, Affiliated Hospital of China Medical University, Shen Yang, 110004, China

**Keywords:** cerebral ischemia reperfusion, lncRNA H19, gene polymorphism, autophagy, apoptosis

## Abstract

Long non-coding RNA H19 (lncRNA H19) was found to be upregulated by hypoxia, its expression and function have never been tested in cerebral ischemia and reperfusion (I/R) injury. This study intended to investigate the role of lncRNA H19 and H19 gene variation in cerebral I/R injury with focusing on its relationship with autophagy activation. Cerebral I/R was induced in rats by middle cerebral artery occlusion followed by reperfusion. SH-SY5Y cells were subjected to oxygen and glucose deprivation and reperfusion (OGD/R) to simulate I/R injury. Real-time PCR, flow cytometry, immunofluorescence and Western blot were used to evaluate the level of lncRNA H19, apoptosis, autophagy and some related proteins. The modified multiple ligase reaction was used to analyze the gene polymorphism of six SNPs in H19, rs217727, rs2067051, rs2251375, rs492994, rs2839698 and rs10732516 in ischemic stroke patients. We found that the expression of lncRNA H19 was upregulated by cerebral I/R in rats, as well as by OGD/R in vitro in the cells. Inhibition of lncRNA H19 and autophagy protected cells from OGD/R-induced death, respectively. Autophagy activation induced by OGD/R was prevented by H19 siRNA. Autophagy inducer, rapamycin, abolished lncRNA H19 effect. Furthermore, we found that lncRNA H19 inhibited autophagy through DUSP5-ERK1/2 axis. The result from blood samples of ischemic patients revealed that the variation of H19 gene increased the risk of ischemic stroke. Taken together, the results of present study suggest that LncRNA H19 could be a new therapeutic target of ischemic stroke.

Ischemic stroke is a serious clinic condition with poor prognosis [1]. Thrombolytic therapy is the only accepted treatment in clinic so far for ischemic stroke, which, however, unavoidably leads to the reperfusion injury. In spite of increasing effort, to deal with reperfusion injury remains a challenge for clinicians. The pathogenesis of ischemic stroke is very complex, involving both environmental and genetic factors, and a series of genetic markers of ischemic stroke have been revealed recently [2].

Long non-coding RNAs (lncRNAs) are a kind of RNAs longer than 200 nucleotides. Since they cannot be translated into proteins, lncRNAs were originally viewed as the noise of translational process. Recent studies revealed that lncRNAs participate in the regulation of protein expression through functioning as the molecular decoys, the mediators of signaling pathways, the molecular guides for transcriptional co-activators and the scaffold for the formation of functional complex [3]. LncRNA H19 expresses mainly in embryo [4]. The abnormal expression of lncRNA H19 has been found in several kinds of tumors such as gastric cancer [5], liver cancer [6], bladder cancer [6] and choriocarcinoma [7]. Recent studies reported that lncRNA H19 re-expresses in the artherosclerotic plaque [8] and animal model of coronary artery disease [9]. Hypoxia is a major cause of cerebral ischemia and reperfusion (I/R) injury, which can stimulate the expression of lncRNA H19 through activating hypoxia induced factor 1α [10]. However, the expression of lncRNA H19 has not been tested in cerebral I/R.

Autophagy activation plays important role in the process of cerebral I/R injury, which is a double-edged sword, its activation will affect the fate of neuron suffered from I/R injury [11,12]. The formation of autophagosome helps clean up the damaged organelles and promote the recycle of energy and materials [13]. However, excessive autophagy activation will digest the substances necessary for maintaining of normal life and induce autophagic cell death including apoptosis [14]. LncRNA H19 may take part in the regulation of autophagy, since studies demonstrated that lncRNA H19 has close relationship with apoptosis [7,10].

H19 gene polymorphism participates in the regulation of lncRNA H19 expression [15], which has been reported to be associated with the risk factors of ischemic stroke, such as coronary artery disease [16], obesity [17], and blood pressure [18]. We thus speculated that the polymorphism of H19 was associated with the risk of ischemic stroke.

In this study, we first employed rat middle cerebral artery occlusion (MCAO) model and human neuroblastoma cell (SH-SY5Y cell line) oxygen glucose deprivation and reperfusion (OGD/R) model to determine whether the level of lncRNA H19 is regulated by I/R challenge and, if yes, whether lncRNA H19 takes part in the regulation of autophagy and what is the underlying mechanism in the process of I/R injury. We next determined H19 gene polymorphism in the blood sample from stroke patients to test the role of H19 gene polymorphism in the regulation of lncRNA H19 expression, since it’s hard to get human brain tissue sample.

## METHODS AND MATERIALS

### Experimental animals and MCAO model

The protocols for all animal experiments were approved by the Institutional Animal Care and Use Committee of China Medical University, and all studies were performed in accordance with principles outlined in the National Institutes of Health Guide for the Care and Use of Laboratory Animals. Temporary focal ischemia was induced in male Sprague-Dawley rats (250-280 g). Following anesthesia with 10% chloral hydrate (350 mg/kg, i.p.), a 4-0 monofilament nylon suture (Beijing Sunbio Biotech Co. Ltd; Beijing, China) with a rounded tip was inserted into the internal carotid artery through the external carotid artery stump, and gently advanced to occlude the middle cerebral artery. After a 120 min middle cerebral artery occlusion (MCAO), the suture was removed to restore blood flow. Sham-operated rats were manipulated in the same manner but without occlusion of the middle cerebral artery. The body temperature was monitored with a rectal probe and maintained at 37 ± 0.5°C with a heating pad and lamp throughout the procedure. All surgical procedures were performed under a stereomicroscope. The rats were sacrificed 24 hours after ischemia and the brains were dissected and sliced into 3-mm thick coronal sections. The sections were then stained with 4% 2,3,5-triphenyltetrazolium chloride (TTC) (Sigma; St. Louis, MO, USA) for 30 minutes, and fixed in 4% paraformaldehyde.

Infarct volume was assessed on 5 slices of 3 mm coronal sections from each brain. The infarct area was estimated by Image J (Bethesda, MD, USA) software. The infarct volume was calculated using a formula: 100 × (contralateral hemisphere volume - non-infarct ipsilateral hemisphere volume) / contralateral hemisphere volume.

### SH-SY5Y cell culture and oxygen and glucose deprivation and reperfusion (OGD/R) treatment

SH-SY5Y cells were purchased from ATCC (Maryland, America). The cells in normal group were cultured in a normal culture medium containing DMEM solution (Gibco/Life Technologies Ltd, Paisley, Scotland) mixed with 10% fetal serum (Gibco/Life Technologies Ltd, Paisley, Scotland) and 7.5% horse serum (Gibco/Life Technologies Ltd, Paisley, Scotland), and incubated in a humidified incubator (Thermo CO_2_ incubator, 311, USA) at 37? and 5% CO_2_. The cells in OGD group were cultured in an ischemia-mimetic solution (mmol/L: 140 NaCl, 3.5 KCl, 0.43 KH2PO_4_, 1.25 MgSO_4_, 1.7 CaCl_2_, 5 NaHCO_3_, 20 HEPES, pH 7.2-7.4) and kept in a hypoxic incubator chamber (Billups- Rothenberg) filled with 95% N_2_/5% CO_2_ at 37? for 4, 8, or 12 hr. Ten percent of intralipid solution at a concentration of 50μM was used as a vehicle (Sigma, St Louis, MO). Rapamycin (RAP) (100 nm; Sigma, R0395) and 3-methyladenine (3-MA) (400 nmol; Sigma, M9281) were used as autophagy activator and inhibitor, respectively, and added to the medium 10 min before OGD.

After OGD for different time, the cells were transferred to normal culture medium and kept in an incubator with 5% CO_2_ at 37?for 24 hr for reperfusion and all the following experiments.

### Cell viability assessment

The viability of SH-SY5Y cells was determined by CCK-8 cell viability test kit (Dojindo, Japan, CK04). Twenty μl CCK-8 solution was added to the culture medium per well. The absorbance value (A) was measured at 450 nm using a spectrophotometer (Thermo, multiskan FC, USA). The percentage of cell viability was calculated using the following formula: cell viability (%) = (A of experiment well/A of control well) x 100%.

### Transfection of cells with H19 siRNA and DUSP5 siRNA

SH-SY5Y cells were transiently transfected with small interference RNA (siRNA) against H19 or DUSP5 (GenePharma, Shang Hai, China.) using Lipofectamine 2000 (Invitrogen, Carlsbad, CA, USA). Briefly, after cultured in normal culture medium for 24 hr, the culture medium was replaced by normal culture medium plus 200 μl siRNA/Lipofectamine 2000 complex (10 μl siRNA, 10 μl Lipofectamine 2000 and 180 μl normal medium) and cells were incubated in a humidified incubator for 24 hr.

### Real-time PCR quantitative analysis

Total RNA from the control and treated cells was isolated using Trizol reagent (Takara, Dalian, Liaoning Province, China). The same method was applied for isolation of total RNA from rat brain tissue which was collected from infarct surrounding area. The primer sequences used for real-time reverse transcription-PCR were as follows: The H19 upstream primer 5′-TCCCAGAACCCACAACA TGAA-3′ and reverse 5′-TTCACCTTCCAGAGCCG ATTC-3′ were used to amplify a 150 bp product; The GAPDH forward primer 5′-CTCCCATTCCTCCACC TTTG-3′, and downstream primer 5′-CCACCA CCCTGT TGCTGTAG-3′ were used to amplify a 110 bp product; The 18s forward primer 5′-CATCTCCTCCCCTATT GCCT-3′, and downstream primer 5′-CCCACACCCCT GTGTGTAGT-3′ were used to amplify a 108 bp product. RNA quantities were determined by a spectrophotometer (NanoDrop 2000, Peqlab, Erlangen, Germany). Total RNA (1.0 μg) was transcribed to cDNA using Reverse Transcriptase M-MLV and an oligo dT primer (Takara, Dalian, China). Quantitative real-time PCR was performed with SYBR premix Ex Taq (Takara) using the Rotorgene 3000 system (Corbett, Sydney, Australia).

### Flow cytometry analysis

Flow cytometry analysis was used to assess the percentage of apoptosis cells in different groups. The cells were stained with Annexin V/PI double staining kit (DOJINDO, Japan; AD10) according to the manufacturer’s instructions.

### Immunofluorescence

Immunofluorescence was used to evaluate the distribution and expression of LC3-II, Beclin1, and P62 in SH-SY5Y cells from normal control and OGD/R groups. For this purpose, the cells were incubated with antibodies against LC3 (1:800; Novus; NB600-1384), Beclin1 (1:100; Abcam Cat# ab55878) and P62 (1:100; Abcam Cat# ab91526), respectively, in a humidified container at 4°C for 12 hr. The cells were rinsed in PBS for 3 times, and incubated with TRITC conjugated anti-rabbit IgG (1:100, Proteintech) at room temperature for 4 hr. 4, 6-diamidino-2-phenylindole (DAPI, 0.0001%, Sigma) was applied to stain nuclei. The cells were examined by a laser confocal microscope (Nikon D-Eclipse C1, Japan).

### Western Blot

Total protein was extracted using a kit (KGP250; Nanjing Keygen Biotech Co. Ltd., Nanjing, China). Whole cell lysat was separated by 10-15% SDS-PAGE then transferred to a nitrocellulose membrane. The membrane was blocked with 5% skimmed milk powder in TBST (0.1% Tween 20 in TBS) for 1 hr at room temperature and incubated overnight at 4°C with antibodies against LC3II (1:500; Abcam, ab62721), Beclin1(1:1000; Abcam, ab55878), P62 (1:1000; Abcam, ab91526), DUSP5 (1:400; Abcam, Cat# ab54939 ), ERK1/2 (1:700; CST, Cat# 4695), p-ERK1/2 (1:1000; CST, Cat# 4370) and GAPDH (1:2000; Santa Cruz Biotechnology; Santa Cruz, CA, USA), followed by incubation with horseradish peroxidase-conjugated goat anti-rabbit IgG antibody (1:3000; Proteintech Group, Inc., Hubei, China). Immunoreactive bands were visualized using a chemiluminescence kit (ECL kit; Santa Cruz Biotechnology, USA), and protein bands were scanned using Chemi Imager 5500 V2.03 software. The integrated density value (IDV) for each band was calculated with a computer-aided image analysis system (Fluor Chen 2.0). The IDV of LC3II was normalized with the IDV of LC3I, while the other proteins were normalized with the IDV of GAPDH.

### Genotyping of H19 gene polymorphism in stroke patients

We recruited 152 patients with ischemic stroke and 150 age-and-gender-matched healthy controls from Shengjing hospital, Affiliated Hospital of China Medical University. The standards of diagnosis of ischemic stroke included that the patients came to hospital with clinical symptoms and signs of focal or global cerebral function loss, and the MRI examination showed newly occurred cerebral infarction in the clinically relevant cerebral areas. The characteristics of the patients were listed in table 1. The DNA was obtained from the blood samples of the subjects using blood DNA extraction kit (TLANamp Blood DNA kit, Dp-318). The six known single nucleotide polymorphisms (SNPs) in H19, rs217727, rs2067051, rs2251375, rs492994, rs2839698 and rs10732516, were obtained through searching in the HapMap database. The polymorphisms were tested using the method of improved multiple ligation detection reaction (iMLDR). The primers were designed as follows: rs217727F: CCGTCTCCACAACTCCAACCAG; rs217727R: CCA GACCTCATCAGCCCAACAT; rs2067051F: GGGCA TACAGCGTCACCAAGTC; rs2067051R: ACCTCACC CACCGCAATTCAT; rs2251375F: TCCAGCACACGT CTCTCTCACC; rs2251375R: CCCACCCCTACTCT CCAGGAAC; rs2839698F: CCCTTCTTTCCAGCCC TAGCTC; rs2839698R: TAACGGGGGAAACTGGGG AAGT; rs4929984F: TGGGGTCCAAGTCATGACCA CT; rs4929984R: GAGGCGGTTTCACCAGGAGAAC; rs10732516F: GGTGGAACACACTGTGATCATCACA TAA; rs10732516R: GAACAATGAGGTGTCCCAGTT GCA. After multiple cycles of PCR reactions and ligase reactions, the data were collected by ABI3730XL sequencer and analyzed by GeneMapper4.1 (AppliedBiosystems, USA). This process is operated by Genesky Biotechnologies Inc., Shanghai, China.

**Table1:** Characteristics of study subjects

Characteristics	Cases(n=152)	Controls(n=150)	*p* value
Age (years)	64.07 ± 11.81657	63.68 ±9.534337	0.7657
Sex (male/female)	107/45	100/50	0.4854
BMI (kg/m2)	24.58 ± 3.014734	23.17 ±3.663666	0.0008
Smoking, n (%)	32.2	16.7	< 0.0001
Drinking, n (%)	17.1	19.3	0.6159
SBP (mmHg)	156.5 ± 26.34424	128.7 ± 10.91012	< 0.0001
DBP (mmHg)	96.32 ± 20.7784	84.93 ± 7.495738	< 0.0001
TC (mmol/L)	4.619 ± 1.230424	4.780 ± 0.980209	0.2557
TG (mmol/L)	2.181 ± 2.04464	1.627 ± 1.785428	0.0092
HDL-C (mmol/L)	1.048 ± 0.273142	1.144 ± 0.289942	0.0069
LDL-C (mmol/L)	3.102 ± 1.040202	2.553 ± 0.790455	< 0.0001
FBG (mmol/L)	5.848 ± 2.253664	5.191 ± 1.080911	0.0055
Hypertension, n (%)	97	12	< 0.0001
Diabetes, n (%)	10	4	0.2004
Hyperlipidaemia, n (%)	64	57	0.5589

Abbreviations: BMI, body mass index; SBP, systolic blood pressure; DBP, diastolic blood pressure; TC, total cholesterol; TG, triglyceride; HDL-C, high-density lipoprotein cholesterol; LDL-C, low-density lipoprotein cholesterol; FBG, fasting blood glucose.

### Statistical Analysis

All data were expressed as the mean value ± SD. Data were analyzed by one-way analysis of variance (ANOVA) followed by the Bonferroni test for multiple comparison. P-values < 0.05 were considered statistically significant. For the analysis of gene polymorphism, the categorical variables were compared using chi-square test. The Hardy-Weinberg equilibrium was evaluated by chi-square goodness-of-fit test. The additive model, dominant model and recessive model were used to compare the difference in genotype distribution between patients and controls. The strength of association between H19 polymorphism and the risk of ischemic stroke was evaluated by odds ratio (OR) and 95% confidence interval (CI).

## RESULTS

### LncRNA H19 expression is upregulated by cerebral I/R and cellular OGD/R

**Figure 1.**
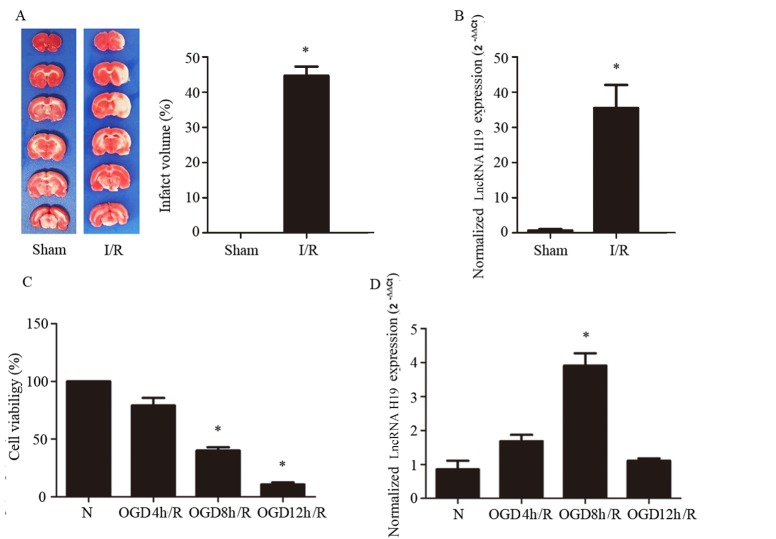
**LncRNA H19 expression in rat cerebral ischemia reperfusion model (I/R) and cellular oxygen glucose deprivation/reperfusion (OGD/R) model. A)** 2, 3, 5-triphenyltetrazolium chloride (TTC) staining evaluation of rat cerebral infarct volume induced by I/R (n=6). The representative images of rat brain slices in different groups are displayed on the left and the quantification of infarct volume on the right. **B)** LncRNA H19 level determined by real-time PCR, normalized to the expression of GAPDH in the sham and I/R group (n=6). **C)** Cell viability induced by different conditions of OGD/R. **D)** LncRNA H19 level determined by real-time PCR, normalized to the expression of 18S rRNA in the normal and OGD/R groups. N represents normal group, OGD/R 4 hr, OGD/R 8 hr, and OGD/R 12 hr represent s the cells subjected to 4, 8, and 12 hr oxygen glucose deprivation, respectively, followed by 24 hr reperfusion. Bar represents the mean value ± SD. *p < 0.05 vs. the sham-operated or normal group.

The change in lncRNA H19 expression after cerebral I/R has never been tested. To gain insight into this issue, we first examined it in a rat MCAO model. The cerebral I/R injury was proved by TTC staining, as shown in Figure 1A, wherein the TTC stained brain slices from different groups are displayed on the left while quantification of infarct volume on the right. The level of H19 RNA accessed by Real-time PCR showed that, in sham group, the level of lncRNA H19 was very low, while cerebral I/R induced ~35 fold-increase in its expression (Fig. 1B). We supposed that lncRNA H19 worked in neuron to exert its effect in cerebral I/R. Therefore, to verify this result and explore the underlying mechanism, we built up a cellular OGD/R model using SH-SY5Y, a neuroblastoma cell line, to mimic cerebral I/R injury. The results of CCK8 showed that 8 hr OGD and 24 hr reperfusion decreased the cell viability to about 40% with significant difference from normal control group (Fig. 1C; *p*<0.05). Importantly, OGD 8 hr/reperfusion 24 hr induced a significant higher expression of lncRNA H19 compared with the normal control group (Fig. 1D; *p*<0.05), which well matched the decrease in cell viability and was in line with the result from in vivo study in MCAO model. In contrast, 12 hr OGD and 24 hr reperfusion further decreased the cell viability to about 20% (Fig. 1C), albeit, but had not significant impact on the expression of lncRNA H19 (Fig.1D), suggesting that some mechanism was turned on in this condition, which either did not involve lncRNA H19 or counteracted the increase in lncRNA H19 expression. Thus, we chose OGD 8 hr/reperfusion 24 hr for the following experiments to gain insight the detail of the mechanism whereby lncRNA H19 regulated cell death.

**Figure 2.**
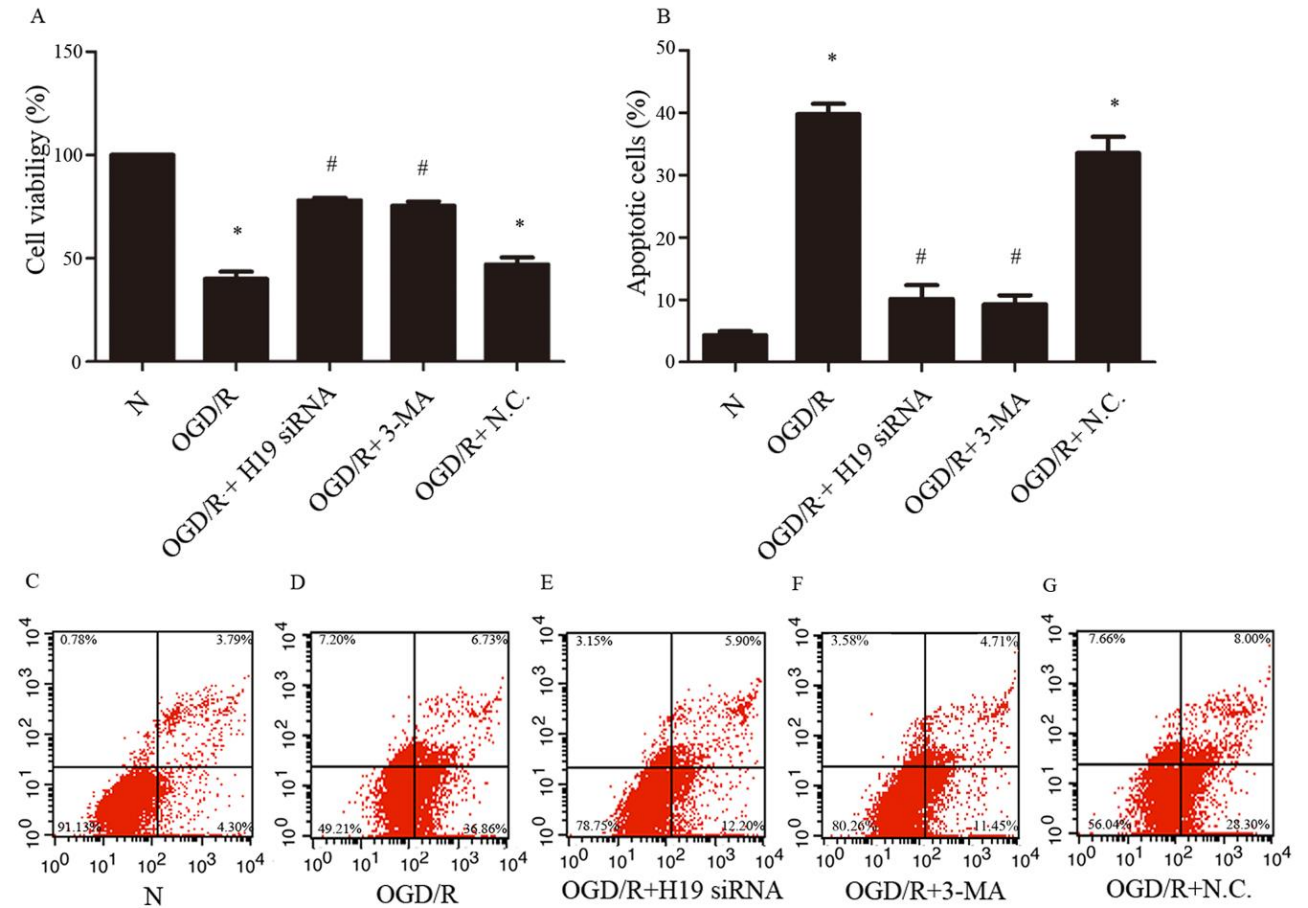
**The effect of inhibition of lncRNA H19 and autophagy on cell viability and apoptosis in OGD/R model. A)** The cell viability in different groups. **B)** Percentage of apoptotic cells in different groups. **C-G)** The result of Annexin V/PI staining in different groups measured by flow cytometry. N, normal group; OGD/R, OGD 8 hr and reperfusion 24 hr; OGD/R + H19 siRNA and OGD/R + N.C, transfecting the cells with H19 siRNA and normal control siRNA, respectively, before OGD/R treatment. OGD/R+3MA, 3MA was added to the medium 10 min before OGD/R. Bar represents the mean value ± SD. *p<0.05 vs. the normal group, ^#^*p*<0.05 vs. OGD/R group, ^&^*p*<0.05 vs. OGD/R + H19 siRNA group.

### LncRNA H19 upregulation and autophagy activation induce cell apoptosis after OGD/R

To evaluate the effect of lncRNA H19 upregulation and autophagy activation on the cells subjected to OGD/R, we used H19 siRNA and 3-methyladenine (3-MA) to inhibit LncRNAH19 and autophagy, respectively. We found that the attenuation of lncRNA H19 expression or autophagy protected the decrease in cell viability induced by OGD/R (Fig. 2A; *p*<0.05). Flow cytometric analysis confirmed this result, showing that OGD/R increased the percentage of apoptotic cells (~40%) significantly compared with control (~4%), while H19 siRNA and 3-MA treatment decreased the percentage of apoptotic cells to ~10% and ~9%, respectively (Fig. 2B-F).

### The upregulation of lncRNA H19 activates autophagy during OGD/R

**Figure 3.**
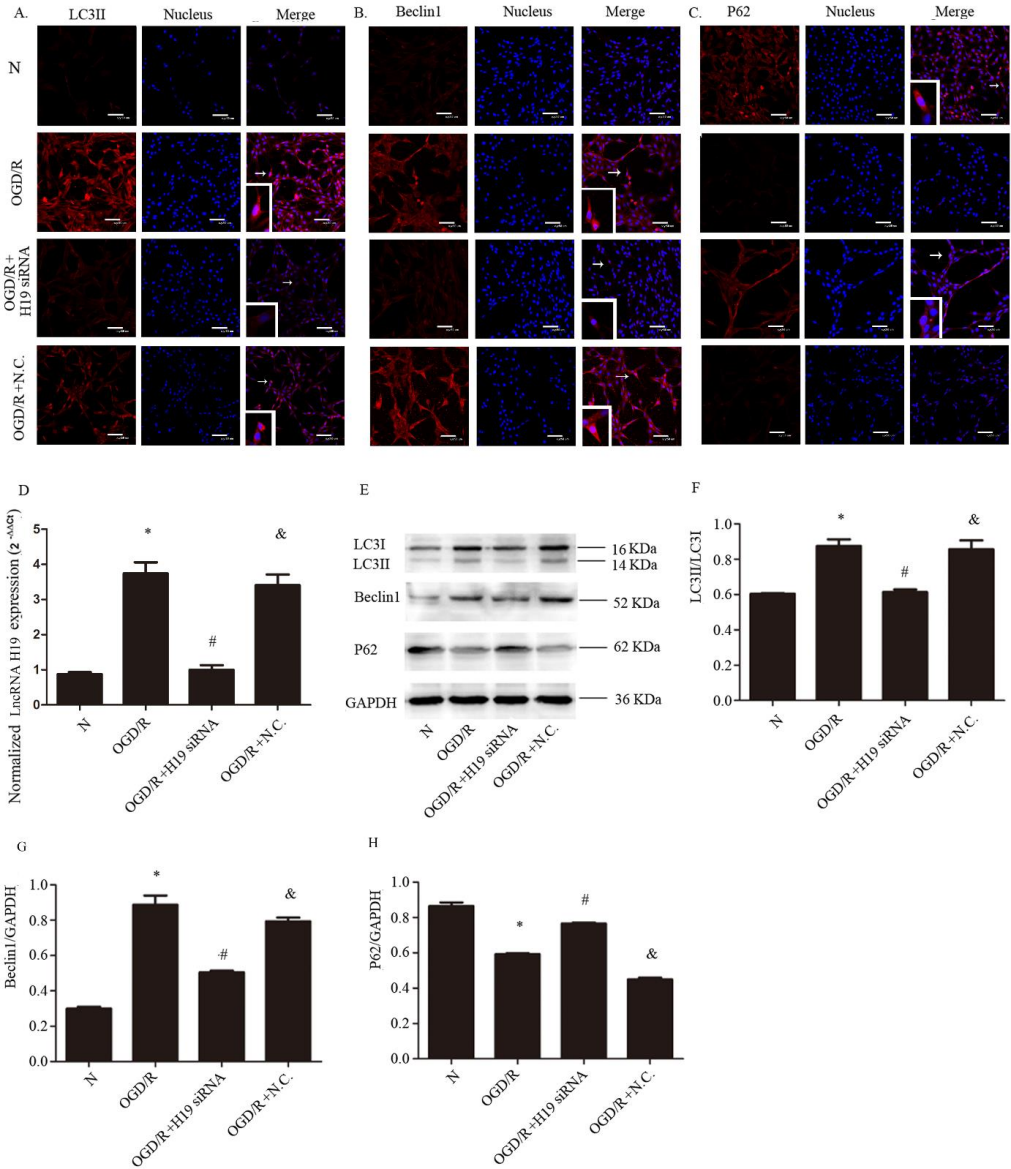
**The effect of H19 siRNA on autophagy in OGD/R model. A)** Immunofluorescence of LC3II in different conditions. **B)** Immunofluorescence of Beclin1 in different conditions. **C)** Immunofluorescence of P62 in different conditions. **D)** The change of lncRNA H19 expression level induced by H19 siRNA. E. Western blot of LC3II, LC3I, Beclin1, and P62 in different groups. **F, G, and H)** Statistical analysis of the expression of LC3II, Beclin1 and P62, respectively. N, normal group; OGD/R, OGD 8 hr and reperfusion 24 hr; OGD/R + H19 siRNA and OGD/R + N.C, transfecting the cells with H19 siRNA and normal control siRNA, respectively, before OGD/R treatment. Bar represents the mean value ± SD. *p<0.05 vs. normal group, ^#^*p*<0.05 vs. OGD/R group, ^&^*p*<0.05 vs. OGD/R + H19 siRNA group. White arrows indicate the co-localized positive cells.

In order to assess the link between lncRNA H19 upregulation and autophagy activation, we employed immunofluorescence and Western blot to evaluate the level of autophagy-related proteins. The results showed that the immunofluorescence of LC3II and Beclin1 were increased by OGD/R, and H19 siRNA prevented their increase (Fig. 3A, B). In contrast, the immunofluorescence of P62 was decreased by OGD/R, while this decrease was inhibited by H19 siRNA (Fig. 3C), indicating that lncRNA H19 promotes autophagy by activating autophagosome formation rather than inhibiting autophagosome degradation. Consistent with immunofluorescence, Western blot showed that the levels of LC3II and Beclin1 were upregulated by OGD/R, which was blunted by H19 siRNA (Fig. 3E-G; *p*<0.05). The change of P62 level in different groups was opposite to that of LC3II and Beclin1 (Fig. 3H; *p*<0.05). The silencing efficiency of lncRNA H19 was tested by real-time PCR and the result is shown in Fig. 3D (*p*<0.05).

**Figure 4.**
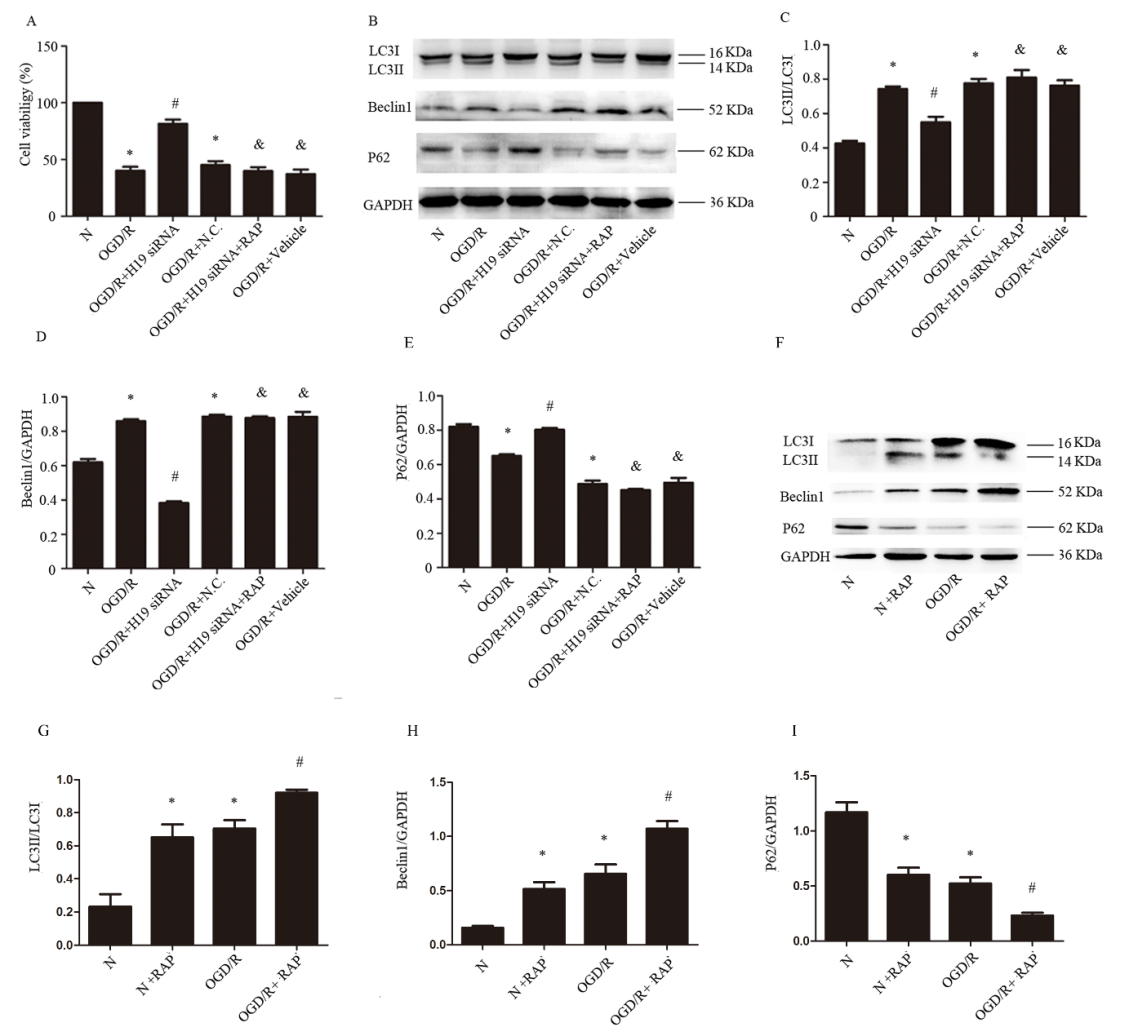
**LncRNA H19 impairs cells in OGD/R via activation of autophagy. A)** The cell viability in different conditions. **B)** Western blot of LC3I, L3II, Beclin1, and P62 in different groups. **C, D** and **E)** Statistical analysis of the expression of LC3II, Beclin1, and P62. **F)** Western blot of LC3I, L3II, Beclin1, and P62 in different groups with or without rapamycin treatment. **G) H and I)** Statistical analysis of the expression of LC3II, Beclin1, and P62. N, normal group; OGD/R, OGD 8 hr and reperfusion 24 hr; OGD/R + H19 siRNA and OGD/R + N.C, transfecting the cells with H19 siRNA and normal control siRNA, respectively, before OGD/R treatment; OGD/R + H19 siRNA + RAP, the cells transfected with H19 siRNA and administration of rapamycin before OGD/R; OGD/R + vehicle, treating the cells with vehicle before OGD/R. Bar represents the mean value ± SD. *p<0.05 vs. the normal group, ^#^*p*<0.05 vs. OGD/R group, ^&^*p*<0.05 vs.OGD/R + H19 siRNA group.

### LncRNA H19 impairs cells viability during OGD/R via activation of autophagy

To determine whether lncRNA H19 impairs cells through activation of autophagy, we used autophagy inducer, rapamycin (RAP), to activate autophagy process. The results of CCK analysis showed that H19 siRNA augmented the percentage of living cells. However, administration of RAP together with H19 siRNA abolished the protective effect of H19 siRNA (Fig. 4A; *p* < 0.05). The results of Western blot showed that H19 siRNA decreased the level of LC3II and Beclin1 but increased the level of P62 (Fig. 4B-E; *p*< 0.05). However, RAP administration activated autophagy in the presence of H19 siRNA (Fig. 4B-E; *P* < 0.05). In order to test the efficiency of RAP in activating autophagy, we compared the levels of autophagy related proteins with or without RAP treatment. The results showed that RAP could activate autophagy in both normal and OGD circumstances (Fig. 4 F-I; *p* < 0.05).

**Figure 5.**
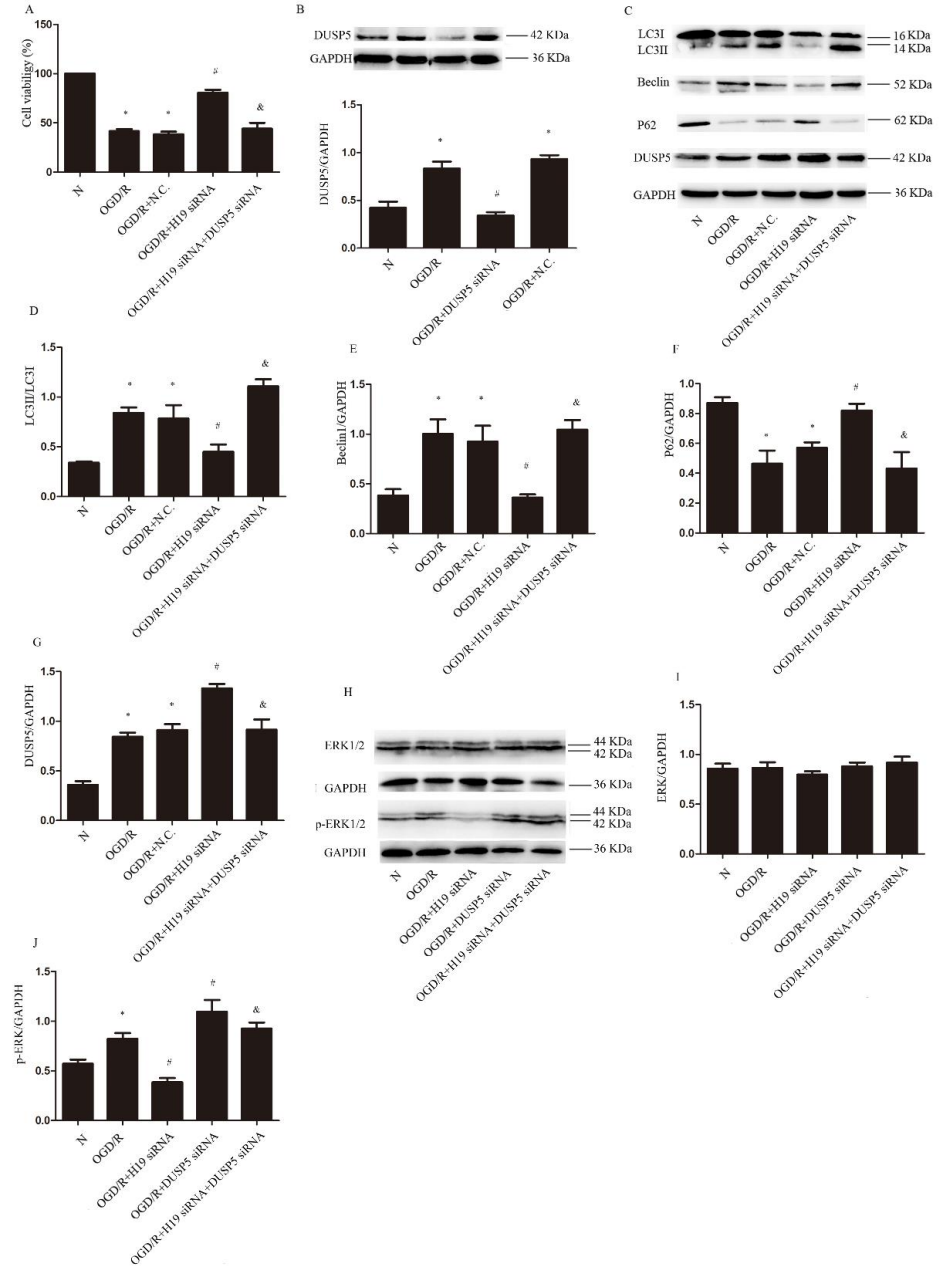
**LncRNA H19 induces autophagy through DUSP5-ERK1/2 axis. A)** The cell viability in different conditions. **B)** Western blot and statistical analysis of DUSP5 in different conditions. **C)** Western blot of LC3I, L3II, Beclin1, P62 and DUSP5 in different groups. **D-G)** Statistical analysis of the expression of LC3II, Beclin1, P62 and DUSP5 in different groups. **H)** Western blot of ERK1/2 and p-ERK1/2 in different groups. **I and J)** Statistical analysis of the expression of ERK1/2 and p-ERK1/2 in different groups. N, normal group; OGD/R, OGD 8 hr and reperfusion 24 hr; OGD/R + H19 siRNA, OGD/R + N.C and OGD/R+DUSP5, transfecting the cells with H19 siRNA, normal control siRNA and DUSP5 siRNA, respectively, before OGD/R treatment; OGD/R + H19 siRNA + DUSP5 siRNA, the cells transfected with H19 siRNA and DUSP5 siRNA before OGD/R. Bar represents the mean value ± SD. *p < 0.05 vs. the normal group, ^#^*p* 0.05 vs. OGD/R group, ^&^*p*<0.05 vs.OGD/R + H19 siRNA group.

**Table 2:** Genotype distribution and allele frequency of the six tested SNPs

Polymorphisms	Cases	Controls	*P* value	Crude OR (95% CI)	*p* value	Adjusted OR^§^(95% CI)	*p* value
rs217727							
Additive model							
CC	18	47		1	-	1	-
CT	63	62		2.653(1.390-5.066)	0.0027	2.381 (1.133-5.005)	0.022
TT	71	41	< 0.001	4.522(2.324-8.799)	< 0.0001	4.288(2.002-9.181)	<0.001
Dominant model							
CC	18	47		1	-	1	-
CT+TT	134	103	0.0571	3.397(1.862-6,196)	< 0.0001	3.081(1.563-6.072)	0.001
Recessive model							
CC+CT	81	109		1	-	1	-
TT	71	41	< 0.001	2.33(1.442-3.767)	0.0005	2.269(1.344-3.828)	0.002
Allele							
C	99	156					
T	205	144	< 0.001				
rs2067051							
Additive model							
TT	28	30		1	-	1	-
TC	54	58		0.9975(0.5288-1.882)	0.9939	1.605(0.529-2.144)	0.861
CC	70	62	0.7105	1.21(0.659-2.245)	0.5459	1.359(0.659-2.801)	0.406
Dominant model							
TT	28	30		1	-	1	-
TC+CC	124	120	0.7276	1.107(0.6242-1.964)	0.7276	1.127(0.648-2.286)	0.542
Recessive model							
TT+TC	82	88		1	-	1	-
CC	70	62	0.4084	1.212(0.7684-1.911)	0.4084	1.319(0.808-2.152)	0.268
Allele							
T	110	118					
C	194	182	0.4247				
rs2251375							
Additive model							
CC	63	72		1	-	1	-
CA	69	57		1.383(0.8498-2.252)	0.1912	1.530(0.900-2.602)	0.116
AA	20	21	0.416	1.088(0.5407-2.191)	0.8123	1.257(0.581-2.718)	0.562
Dominant model							
CC	63	72		1	-	1	-
CA+AA	89	78	0.2521	1.304(0.8275-2.055)	0.2521	1.392(0.852-2.273)	0.186
Recessive model							
CC+CA	132	129		1	-	1	-
AA	20	21	0.8308	0.9307(0.4816-1.799)	0.8308	0.973(0.474-1.997)	0.941
Allele							
C	195	201					
A	109	99	0.4603				
rs4929984							
Additive model							
CC	40	68		1	-	1	-
CA	62	53		1.989(1.164-3.398)	0.0115	2.275(1.251-4.138)	0.007
AA	50	29	0.0305	2.931(1.606-5.350)	0.0004	3.020(1.531-5.959)	0.001
Dominant model							
CC	40	68		1	-	1	-
CA+AA	112	82	0.0006	2.322(1.432-3.766)	0.0006	2.506(1.473-4.262)	0.001
Recessive moedel							
CC+CA	102	121		1	-	1	-
AA	50	29	0.0073	2.045(1.206-3.468)	0.0073	1.942(1.088-3,464)	0.025
Allele							
C	142	189					
A	162	111	< 0.001				
rs2839698							
Additive model							
GG	80	87		1	-	1	-
GA	61	50		1.327(0.8195-2.148)	0.2495	1.563(0.925-2.641)	0.095
AA	11	13	0.4637	0.9202(0.3899-2.172)	0.8494	1.086(0.673-1.752)	0.736
Dominant model							
GG	80	87		1	-	1	-
GG+GA	72	63	0.3481	1.243(0.7889-1.958)	0.3481	1.346(0.826-2.195)	0.233
Recessive model							
GG+GA	141	137					
AA	11	13	0.646	0.8221(0.3560-1.898)	0.646	0.665(0.262-1.690)	0.391
Allele							
G	221	224					
A	83	76	0.5827				
rs10732516							
Additive model							
GG	69	62		1	-	1	-
GA	45	47		0.8603(0.5045-1.467)	0.5804	0.768(0.419-1.409)	0.394
AA	38	41	0.7717	0.8328(0.4761-1.457)	0.521	0.917(0.497-1.689)	0.78
Dominant model							
GG	69	62		1	-	1	-
GA+AA	83	88	0.4764	0.8475(0.5373-1.337)	0.4764	0.822(0.499-1.353)	0.441
Recessive model							
GG+GA	114	109		1	-	1	-
AA	38	41	0.6446	0.8862(0.5302-1.481)	0.6446	1.060(0.610-1.843)	0.836
Allele							
G	183	171					
A	121	129	0.425				

The bold font means the value having significant difference with *p*<0.05

Abbreviations: OR, odds radio; CI, confidence interval.

§ Adjusted for age, gender, smoking status, drinking status, body mass index, hypertension, diabetes, total cholesterol, triglyceride, high-density lipoprotein cholesterol, low-density lipoprotein cholesterol.

### LncRNA H19 inhibits DUSP5-ERK1/2 axis inducing autophagy activation

To determine whether lncRNA H19 activated autophagy through DUSP5, we used DUSP5 siRNA to inhibit DUSP5 expression. The results of cell viability assessment showed that the addition of DUSP5 siRNA abolished the protective effect of H19 inhibition on cell viability (Fig. 5A; *p* < 0.05). The efficiency of DUSP5 siRNA is shown in Fig. 5B (*p*<0.05). Western blot revealed that DUSP5 was upregulated by OGD/R and further increased by H19 siRNA (Fig. 5C and G; *p* < 0.05). The levels of LC3II and Beclin1 increased in response to OGD/R, which was prevented by LncRNA H19 inhibition, while this preventive effect of LncRNA H19 inhibition was abolished by the addition of DUSP5 siRNA (Fig. 5C-E; *p* < 0.05). The level of P62 varied among groups in a manner opposite to LC3II and Beclin1 (Fig.5 C and F; *p* < 0.05). Taken together, these results indicated DUSP5 as the downstream effector of lncRNA H19 in the process of autophagy activation. The level of total ERK1/2 was not changed by OGD/R challenge, nor by the treatment of either H19 siRNA or DUSP5 siRNA (Fig. 5 H, I. *p* > 0.05). Whereas, p-ERK1/2 was increased significantly by OGD/R stimulation, which was prevented by H19 siRNA, while augmented by DUSP5 siRNA, irrespective of the presence of H19 siRNA or not (Fig.5 H and J; *p*<0.05).

### H19 gene polymorphism and the morbidity of ischemic stroke

H19 gene polymorphism in ischemic stroke patients has never been examined. We collected the blood samples from 152 patients with ischemic stroke and 150 healthy controls. The basic information of the stroke patients and healthy controls is presented in Table 1. Age and gender of the subjects in the two groups had no statistical differences, demonstrating that the two groups were well matched. No significant difference was observed either between the two groups in the total cholesterol, morbidity of diabetes and hyperlipidaemia, and the number of subjects having the habit of drinking. On the other hand, compared to control group, ischemic stroke group had larger body mass index, higher level of systolic blood pressure, diastolic blood pressure, triglyceride, low density lipoprotein and fasting blood glucose, lower level of high-density lipoprotein cholesterol, and greater number of subjects who were used to smoking.

The genotype distribution and allele frequency of the six SNPs tested are shown in Table 2. Genotype frequencies did not deviate form Hardy-Weinberg equilibrium in both groups (*p* > 0.05). In all of the six SNPs, only the genotype and allele frequency of rs217727 and rs4929984 had significant differences between ischemic stroke group and control (*p* < 0.05). After adjustment for age, gender, smoking, drinking, BMI, hypertension, diabetes, TC, TG, HDL-C, LDL-C, we found that the subjects with CT or TT genotype of rs217727 had higher risk of stroke compared with those with CC genotype (Additive model, CT: OR=2.381, 95% CI = 1.133-5.005, *p* = 0.022; TT: OR = 4.288, 95% CI = 2.002-9.181, *p* < 0.001), the subjects of TT and CT genotype had 3.081 times higher risk of getting ischemic stroke when compared with the subjects of CC (Dominant model, OR = 3.081, 95% CI = 1.563-6.072, *p*=0.001), and the subjects of TT genotype were 2.269 times higher riskier than those with CC and CT (Recessive model, OR=2.269, 95% CI = 1.344-3.828, *p*=0.002). A significant difference was found between ischemic stroke group and control group in allele frequency of T and C of rs217727 (*p*<0.001). The A allele of rs4929984 was significantly associated with increased risk of stroke compared with C allele (*p*<0.001). After adjustment for age, gender, smoking, drinking, BMI, hypertension, diabetes, TC, TG, HDL-C, LDL-C, we found that subjects with the genotype including A allele had larger risk of stroke (Additive model, CA: OR = 2.275, 95% CI = 1.251-4.138, *p*= 0.007, AA: OR=3.020, 95% CI=1.531-5.959, *p*=0.001; Dominant model, OR=2.506, 95% CI=1.473-4.262, *p*=0.001; Recessive model, OR=1.942, 95% CI = 1.088-3.464, *p*=0.025).

## DISCUSSION

To the best of our knowledge, the expression and function of lncRNA H19 in the process of cerebral I/R have not been reported, although the relationship of lncRNA H19 expression with some cancers has been confirmed [5-7,15]. In the present study, we examined lncRNA H19 in MCAO and reperfusion rats and OGD/R SH-SY5Y cells. The results revealed that the expression of lncRNA H19 was upregulated in both I/R injury models. In cellular OGD/R model, inhibition of lncRNA H19 and autophagy was observed to increase cell viability and decrease the amount of cells undergoing apoptosis. Furthermore, the protective effect of H19 siRNA was abrogated by autophagy activator, RAP, suggesting that lncRNA H19 induced cerebral ischemia and reperfusion injury by activating autophagy process. Finally, Western blot assessment demonstrated that lncRNA H19 activated autophagy through regulating DUSP5-ERK1/2 axis.

The formation of autophagosome during cerebral ischemia reperfusion has been viewed as a protective mechanism, since it could help clean up the damaged organelles and promote the recycle of energy and materials. However, excessive autophagy formation has been reported to induce apoptosis, necrosis and autophagic death of neuron[13, 14]. LncRNA is known to induce autophagy through diverse mechanisms. In this regard, LncRNA is able to target some autophagy protein related microRNAs to regulate autophagy. LncRNA APF regulates miR-188-3p thus affects ATG7 expression to affect autophagy [19]. FLJ11812 could bind to miR-4459 targeting ATG13 to regulate autophagy [20]. LncRNA TGFB2-OT1 has been found to bind to miR-3960, miR-4488 and miR-4459, regulating the expression of the miRNA that targets CERS1, NAT8L, and LARP1, the key proteins involved in autophagy [21]. lncRNA could also target autophagy related signal pathway, such as mTOR and PI3K-AKT, as reported for LncRNA MEG3 [22]. In the present study, we revealed that lncRNA H19 could inhibit DUSP5, a mitogen-activated protein kinase phosphatase. The upregulation of DUSP5 is known to inhibit ERK1/2 and induces autophagy inhibition [23]. The present study showed that DUSP5 was upregulatd by H19 siRNA, a finding similar to the result of H19 inhibition in JAR cell line [24], which implied that DUSP5 was the target of lncRNA H19. Moreover, application of DUSP5 siRNA abolished the autophagy inhibition effect of H19 siRNA, pointing to that H19 siRNA inhibited autophagy through upregulating DUSP5. To further validate the involvement of DUSP5-ERK1/2 axis in lncRNA H19 action, we tested the level of p-ERK1/2 and found that, as expected, it was inhibited by H19 siRNA, and this inhibition effect was abolished by DUSP5 siRNA. These results indicated that lncRNA H19 acts through inhibiting DUSP5.thus activating ERK1/2.

To gain insight into the role of lncRNA H19 in clinic, we examined H19 gene polymorphism in the blood sample of stroke patients, since it is impractical to have human brain samples. We tested six candidate SNPs, which have been reported to be either associated with those diseases that have similar pathogenesis with ischemic stroke, or associated with the risk factors of the ischemic stroke [16-18]. The results showed that the subjects with CT and TT genotype of rs217727 were 3.081 times riskier in getting ischemic stroke. The subjects with TT genotype remained more susceptible to ischemic stroke after adjustment of age, gender, smoking, drinking, BMI, hypertension, diabetes, TC, TG, HDL-C, and LDL-C. Moreover, the subjects with A allele of rs4929984 exhibited larger likelihood in getting ischemic stroke. For rs217727, the C to T variant has been reported to correlate with elevated SBP and mean arterial pressure [18], and the variant induces a 46% increased risk in getting coronary artery disease [16]. In the present study, an elevated blood pressure was observed in the T allele carriers compared with those with C allele. Since blood pressure is one of the major risk factors of ischemic stroke, the effect of rs217727 variation on the morbidity of ischemic stroke may attribute to its regulation to blood pressure. On the other hand, multiple logistic regression analysis showed that C to T variant of rs217727 was independently associated with the risk of ischemic stroke after adjustment of SBP and DBP. Furthermore, the variant of rs217727 has been found to affect the expression of lncRNA H19 [15]. Thus, it is more likely that rs217727 increases the onset of ischemic stroke through regulation of lncRNA H19 expression. Rs4929984 has been reported to be associated with birth weight, a factor that may affect the risk of ischemic stroke. However, the relationship between birth weight and rs4929984 remains to be verified, as studies found that it depends on which parent the infant’s rs4929984 allele inherited from [25]. The mechanism for the regulation effect of rs4929984 variant on the onset of ischemic stroke needs further research.

## CONCLUSION

The present study demonstrated, for the first time, the upregulation of expression of lncRNA H19 in the brain tissue of rat suffered from cerebral I/R injury. Experiment in cell model showed that LncRNA H19 impaired cells viability by activating autophagy process through a mechanism involving DUSP5-ERK1/2 axis. Analysis of H19 gene polymorphism in the blood sample of stroke patients revealed that C to T variation of rs217727 and C to A variation of rs4929984 in H19 gene increased the risk of ischemic stroke. LncRNA H19 is therefore a potential genetic marker and therapeutic target of ischemic stroke. However, there exist some limitations in our research. Firstly, the mechanisms for the lncRNA H19 detrimental effect need to be elucidated by further study. Secondly, a new animal model remains to be established to test the effect of H19 siRNA in vivo, since delivering siRNA to the rat brain in the present setting costs too much to apply. In addition, SH-SY5Y used in the present study is an immortal cells line, which may differ from primary cultured neurons in some aspect. Thus the result derived from SH-SY5Y cells needs to be verified in future in primary cultured neurons. Finally, in the study of H19 gene polymorphism, the sample size of cases and controls was not large enough, which may result in some bias in the data.
